# Evaluation of a Multipolar MEMS Based Low‐Power Radiofrequency Device for Pain‐Free Facial Rejuvenation: A Prospective Clinical Study

**DOI:** 10.1111/jocd.71258

**Published:** 2026-10-28

**Authors:** Rongwei Mao, Shengshuo Yan, Aditi A. Sharma, Tony Shum, Yang Zhang

**Affiliations:** ^1^ Department of Electrical Engineering and Computer Science University of California, Irvine Irvine California USA; ^2^ Zhejiang‐California International NanoSystems Institute Hangzhou China; ^3^ Department of Dermatology University of California, Irvine Irvine California USA; ^4^ DSC Laser & Skin Care Center San Gabriel California USA

## Abstract

**Background:**

Noninvasive radiofrequency (RF) therapy has emerged as a prominent approach for facial rejuvenation due to its ability to stimulate collagen remodeling without epidermal ablation. However, conventional RF systems often rely on high thermal output, posing risks of heat‐induced dermal damage.

**Objective:**

This study aimed to evaluate the safety and efficacy of a novel micro‐RF device (nebulyft), utilizing a high‐density 321‐electrode MEMS array designed to deliver high precision low‐power phase‐controlled RF energy for facial wrinkle reduction and improved skin texture.

**Methods:**

In this prospective, open‐label clinical trial, 42 participants from California, USA (age 29–62, Fitzpatrick skin types II–IV) completed a 4‐week treatment protocol using the nebulyft device. Each subject underwent one supervised clinic‐based session, followed by at‐home treatments (16 min per session, ≥ 5 sessions per week). Efficacy was assessed using the Fitzpatrick Wrinkle Severity Scale (FWSS) by three blinded board‐certified dermatologists, and subject satisfaction was recorded via a 4‐point Likert scale.

**Results:**

At one‐month post‐treatment visit, 90.5% of participants in the primary FAS (38/42) met the ≥ 1‐point FWSS responder criterion; the supportive PPS result was 92.7% (38/41), as determined by at least two blinded evaluators (FAS exact one‐sided *p* = 0.0015 vs. the prespecified 70% performance goal). The mean subject satisfaction score was 15.76 ± 0.54 out of 16. No pain was reported by any participant during treatment sessions. No adverse events, discomfort, or device malfunctions were reported.

## 1 Introduction

Skin aging is a multifaceted biological process driven by intrinsic factors (e.g., genetic predisposition, hormonal changes, and cellular senescence) and extrinsic factors (e.g., UV exposure, pollution, and lifestyle habits), collectively contributing to structural and functional decline [1]. The aging process is accompanied by phenotypic changes of skin cells and structural and functional changes of extracellular matrix components (such as collagen and elastin). Clinically, aging skin manifests as wrinkles, reduced elasticity, dryness, and uneven pigmentation, significantly impacting both aesthetic concerns and psychological well‐being [2]. As the demand for effective and minimally invasive anti‐aging treatments rises, extensive research has focused on developing therapies that target key aging mechanisms, including collagen degradation, oxidative stress, and diminished cellular turnover [3].

Current anti‐aging interventions encompass topical agents (e.g., retinoids [4], growth factors [5], and antioxidants [6]), injectables (e.g., hyaluronic acid fillers [7], botulinum toxin [8]), and energy‐based devices (e.g., lasers [9], intense pulsed light [10]). While these treatments demonstrate varying degrees of efficacy, they are often limited by drawbacks such as transient results, potential adverse effects (e.g., irritation [11], hypersensitivity [12], or muscle atrophy with neurotoxins [13]), and high maintenance requirements [14]. Moreover, invasive procedures like surgical face‐lifts carry risks of scarring and prolonged recovery, making them less appealing for many patients [15]. Consequently, there is a growing need for safer, longer‐lasting, and noninvasive alternatives.

Among emerging technologies, radiofrequency (RF) therapy has gained prominence as a non‐ablative, energy‐based modality for skin rejuvenation [16]. By delivering controlled thermal energy to the dermis, RF stimulates collagen and elastin production while promoting tissue remodeling, leading to gradual and natural‐looking improvements in skin tightness and texture [17]. Unlike laser treatments, RF is color‐blind and thus suitable for all skin types, with a favorable safety profile and minimal downtime [18]. However, significant thermal damage remains a concern with traditional RF systems since they employ high energy RF generators reaching 100 W level of power. For home everyday purposes, an RF device that can minimize thermal damage and eliminate side effects such as erythema and burns [19], would be greatly appreciated by clinical experts and home users.

The devices studied in this article (nebulyft) utilize a high precision, low‐power radio frequency array manufactured by MEMS (Mircro Electro Mechanical Systems) processes. A total of 321 independent gold‐plated electrodes collectively apply phase interfered RF power to transmit RF energy to the derma layer and accurately heat target cells to 40°C–42°C. MEMS‐enabled medical devices are gaining increasing clinical attention due to their miniature form factor and versatile applications. MEMS‐based medical technologies are increasingly being explored.

The nebulyft device represents a novel high‐precision, low‐power radiofrequency device incorporating a high‐density 321‐electrode RF array, specifically engineered for facial wrinkle reduction. This article presents a single‐arm prospective study on anti‐aging treatment of the skin. By reviewing pre‐clinical and clinical evidence, we aim to evaluate the safety and efficacy of the nebulyft device for clinical and home treatments.

## 2 Materials and Methods

### 2.1 Treatment Device

The nebulyft device served as the skin treatment apparatus in this study as shown in Figure 1.

**FIGURE 1:**
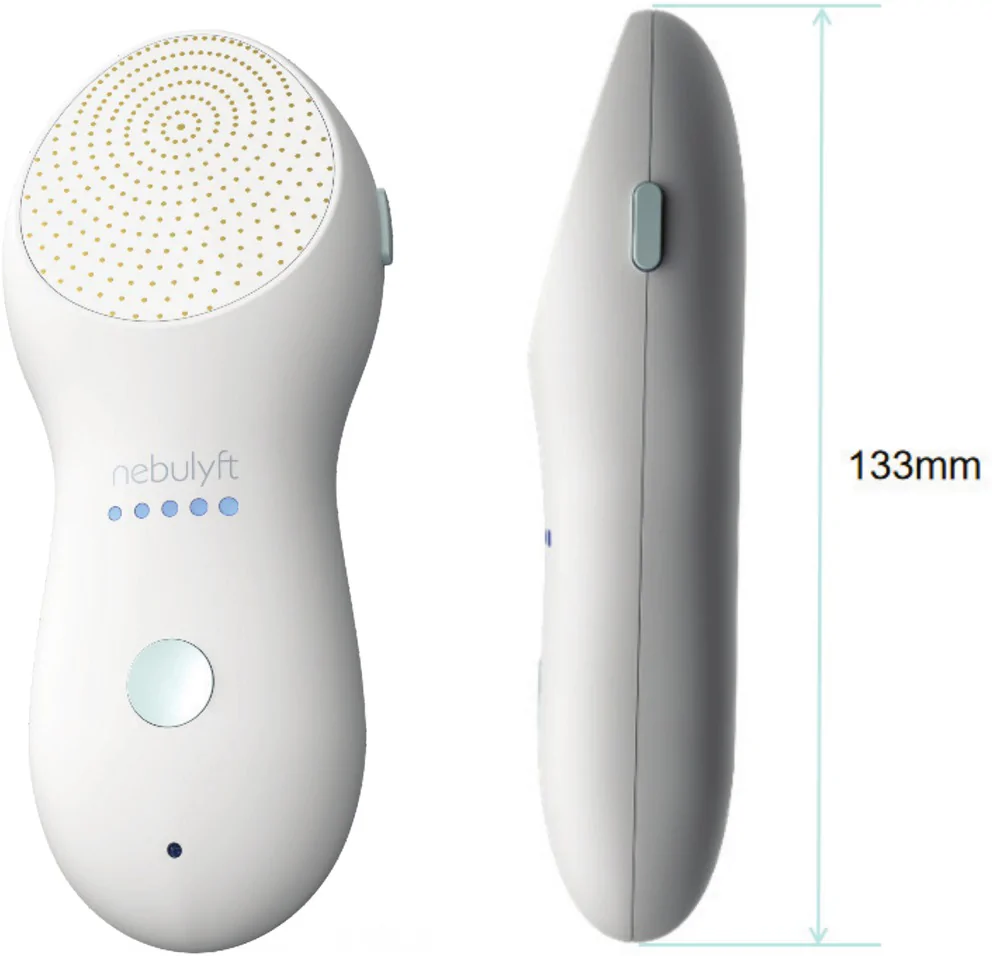
The RFMEMS device nebulyft R1M (RT‐BA‐I) used in the study.

The nebulyft device emits up to 2 W of phase‐controlled RF energy through 321 electrodes on the skin. Independent phase control of individual RF electrodes allows for coherent RF energy flow within the skin and a significant reduction of heat generation.

The energy penetration into the skin is modulated by adjusting the phase relationship between the electrodes. To demonstrate this effect, Figure 2 shows a 90‐degree phase difference applied between the electrodes, and the energy distribution is more concentrated near the skin surface. In contrast, a 0‐degree phase difference between two adjacent electrodes results in deeper penetration of RF energy. All nebulyft electrodes are designed to perform real‐time phase control for most efficient RF energy transmission to the skin's dermal layer.

**FIGURE 2:**
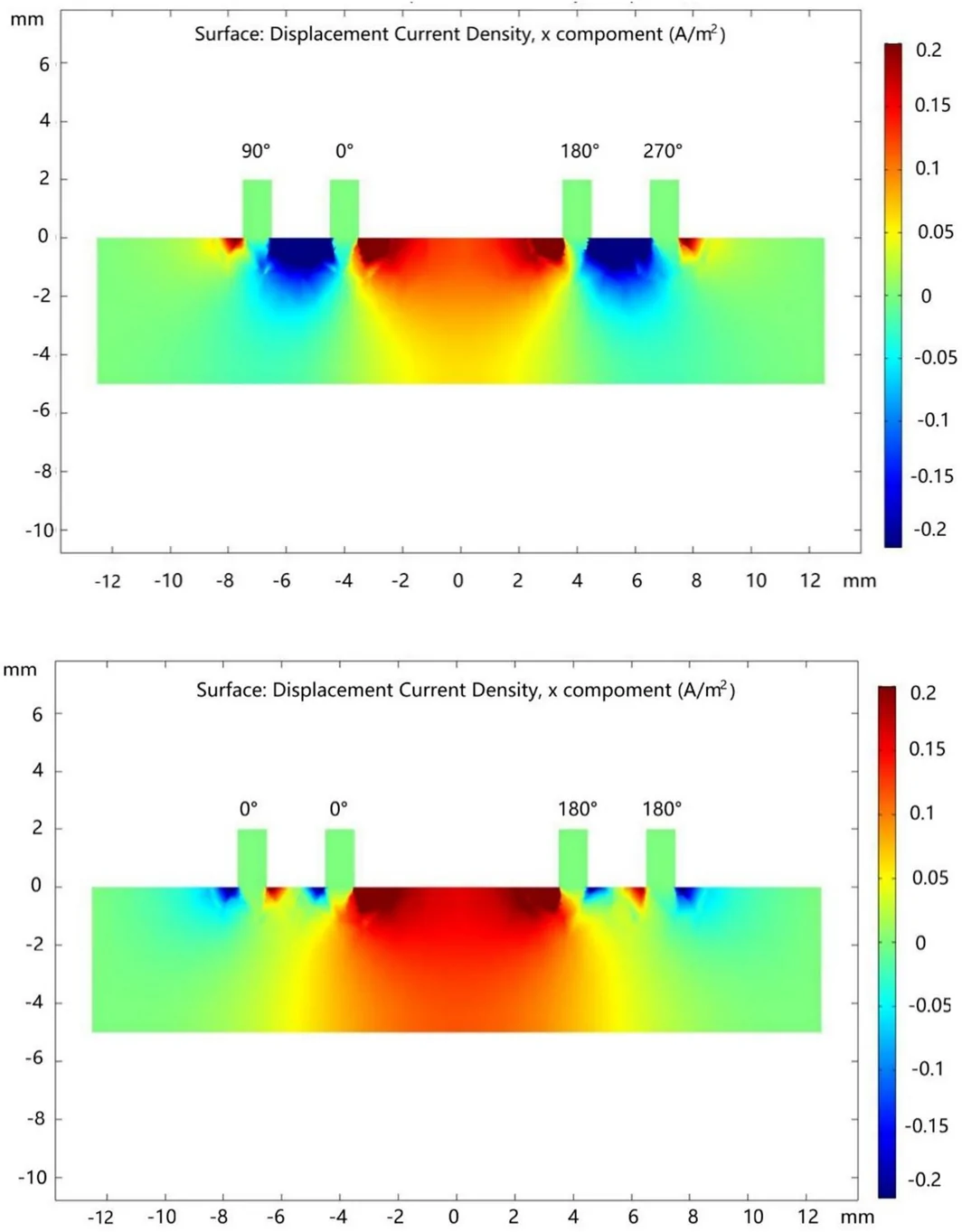
Simulation of the current density distribution with two phase setup of four electrodes.

### 2.2 Study Design and Protocol

This was a prospective, single‐center, open‐label clinical study designed to evaluate the safety and efficacy of a micro‐radiofrequency skin treatment device (nebulyft, Model R1M RT‐BA‐I) for the reduction of facial wrinkles. The study was conducted at DSC Laser & Skin Care Center (San Gabriel, CA) and approved by the Institutional Review Board.

A total of 42 eligible subjects (age range 29–62) were enrolled, of whom 41 completed the full study protocol. Subjects included both males and females (90.5% female) with Fitzpatrick skin types II–IV and visible facial wrinkles. All participants provided written informed consents for participation and photography. Subjects were required to be healthy, able to read the device manual in English, and willing to comply with the study protocol for at‐home device usage and follow‐up visits. Exclusion criteria included active dermatologic conditions in the treatment area, implantable electronic devices, pregnancy or breastfeeding, recent use of isotretinoin or injectable facial treatments, or any condition deemed by the investigator to interfere with study participation or pose undue risk.

The sample size was determined using a normal‐approximation single‐group target‐value method [20], as shown in Equation (1).
(1)

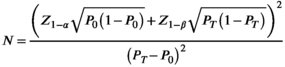

where *N* represents the required evaluable sample size; $P_{0}$ is the pre‐specified objective performance criterion or target efficacy (set at 70%, or 0.70); $P_{T}$ is the expected clinical efficacy rate of the device (set at 95%, or 0.95); $\;Z_{1-\alpha }$ is the standard normal quantile corresponding to a one‐sided significance level of α = 0.025; $\;Z_{1-\beta }$ is the standard normal quantile corresponding to a desired statistical power of 95%, where β = 0.05.

Based on prior clinical expectations for this device class, we set a performance criterion *P*_0_ of 70% and an expected efficacy rate *P*_*T*_ of 95%. With a one‐sided significance level of α = 0.025 (*Z*_1 − α_ = 1.96) and 95% power (β = 0.05; *Z*_1 − β_ = 1.64), substitution into Equation (1) yielded *N* = 25.23, which was rounded up to a minimum evaluable sample size of 26 participants. Considering potential dropouts, a conservative enrollment target of 42 participants was prospectively set in the clinical protocol, exceeding the minimum required sample size and providing greater statistical precision and broader representation of the study population. For the actual primary FAS (*n* = 42), the critical responder threshold for the prespecified one‐sided exact binomial test against *P*_0_ = 0.70 was 36 responders (*P*[X ≥ 36 | *n* = 42, *p* = 0.70] = 0.0155). The anticipated response rate of 95% was supported by a previous study of a comparable home‐use RF device, in which three independent evaluators reported ≥ 1‐point Fitzpatrick‐scale improvement rates of 91.93%, 96.77%, and 98.39%, respectively [21]. The 70% performance goal was selected as a clinically meaningful benchmark consistent with a published RF facial‐rejuvenation study employing three blinded evaluators and a 70% performance criterion [22].

All subjects were instructed in the use of the test device during an initial in‐clinic visit, where the first treatment session was performed under the direct supervision of the principal investigator. Target facial regions included areas around the nasolabial folds and outer canthi (crow's feet). Participants were assigned devices for at‐home treatment and were instructed to self‐administer treatment for 4 min per treatment area at a frequency of ≥ 5 sessions per week for four consecutive weeks. Participants were provided with daily diary forms before the at‐home phase and were instructed to record treatment use and daily observations throughout the 4‐week period. Completed diaries were collected at follow‐up, and treatment frequency was summarized from these records. Remote support from the investigator was available as needed during the at‐home treatment phase. Subjects returned for standardized photography at the end of the treatment period and the 4‐week post‐treatment follow‐up visit. Device usability and labeling comprehension were assessed during the final visit. Subjects were also asked to record any adverse reactions or difficulties experienced during the treatment period.

### 2.3 Study Endpoints and Outcome Measures

The primary efficacy endpoint of the study was the improvement of the FWSS in any treated area, as assessed by a panel of three independent, blinded, and US Board of Dermatology certified dermatologists. The scoring of FWSS is listed in Table 1. All images from patients were recorded and presented electronically. The order of presentation of before and after, as well as the order of presentation of patients was randomized by the statistician's form. Evaluators compared randomized facial photographs taken at baseline and at 1 month following completion of the treatment protocol. A subject was considered improved if at least two of the three evaluators recorded a ≥ 1‐point improvement in FWSS.

**TABLE 1:** Fitzpatrick Wrinkle Severity Scale.

Class	Wrinkling	Score	Degree of elastosis
I	Fine wrinkles (rhytides)	1–3	Mild (fine textural changes with subtly accentuated skin lines)
II	Fine to moderate depth wrinkles, moderate number of lines	4–6	Moderate (distinct papular elastosis [individual papules with yellow translucency under direct lighting] and dyschromia)
III	Fine to deep wrinkles, numerous lines, with or without redundant skin folds	7–9	Severe (multipapular and confluent elastosis [thickened yellow and pallid] approaching or consistent with cutis)

The secondary efficacy endpoint was the subject‐reported satisfaction score, assessed at the end of the treatment period and the 4 week post‐treatment follow‐up visit. Participants completed a standardized questionnaire covering satisfaction with treatment results, safety, ease of use, and ease of learning. Each item was rated on a 4‐point Likert scale, yielding a maximum total satisfaction score of 16.

The safety endpoint included physician‐monitored and subject‐reported adverse events during the study period. Any adverse skin reactions—including erythema, tingling, itching, burning, or edema—were recorded and graded according to standardized dermatologic response criteria. Pain levels during treatment were assessed using a 0–10 Visual Analog Scale (VAS), with ratings collected both at the initial supervised visit and at the final follow‐up evaluation.

### 2.4 Statistical Analysis

Continuous variables, such as age, treatment frequency, and satisfaction scores, were summarized using mean, standard deviation, median, and range. Categorical variables, including sex, Fitzpatrick skin type, and adverse event occurrence, were reported as counts and percentages.

Statistical tests were two‐sided where applicable; a one‐sided significance level of 0.025 was prespecified for the primary endpoint analysis. For the primary efficacy endpoint, an exact binomial test was performed to test the observed responder rate against the pre‐specified objective performance criterion (H0: *p* ≤ 0.70). Two‐sided 95% exact Clopper‐Pearson confidence intervals were calculated for responder rates. Data analysis was conducted in accordance with the intention‐to‐treat (ITT) principle. The full analysis set (FAS), including all 42 enrolled participants, was used as the primary efficacy analysis population. The per‐protocol set (PPS), comprising the 41 participants who completed the protocol and final photographic efficacy assessment, was reported as a supportive analysis. Inter‐rater agreement for the binary responder classification was evaluated using Gwet's first‐order agreement coefficient (AC1), with overall pairwise percent agreement also reported. AC1 was selected because the responder classifications were highly imbalanced toward improvement and this coefficient is less sensitive than conventional kappa statistics to prevalence‐related instability [23].

## 3 Results

A total of 67 subjects were screened for eligibility. Of these, 42 subjects met the inclusion and exclusion criteria and were enrolled in the study. Among the enrolled participants, 41 subjects (97.6%) completed the full treatment protocol and all scheduled follow‐up assessments. One subject, who had completed all scheduled treatment sessions, was unable to attend the final follow‐up visit for photographic documentation due to reasons unrelated to the study and was therefore considered withdrawn from the study. The FAS, used for the primary efficacy analysis, included all 42 enrolled subjects. The PPS included the 41 subjects who completed the study and final photographic assessment without protocol deviations and was used as a supportive analysis. The participant without the final photographic assessment was retained in the FAS and conservatively counted as a non‐responder because the prespecified photographic responder criterion could not be demonstrated. The safety analysis set (SAS) also included all 42 enrolled subjects.

Based on the returned participant diaries, all participants reported adherence to the recommended usage frequency of at least five sessions per week. The mean recorded treatment frequency was 28 sessions over the 4‐week period (SD 3; median 28; range 22–32 sessions).

Of the 42 enrolled participants, 90.5% (*n* = 38) were female and 9.5% (*n* = 4) were male. The mean age was 47.0 ± 8.0 years (range: 29–62 years). The racial distribution included 76.2% Asian (*n* = 32), 14.3% Hispanic (*n* = 6), and the remaining White/Caucasian (*n* = 4). All subjects had visible facial wrinkles and Fitzpatrick skin types II–IV, as required by the inclusion criteria. Baseline vital signs, including blood pressure, heart rate, and body temperature, were within normal physiological ranges across all participants.

The primary efficacy endpoint—defined as an improvement of at least one point of FWSS in at least one treated area—was met by 90.48% (38/42) of subjects in the primary FAS analysis and 92.68% (38/41) in the supportive PPS analysis, as listed in Table 2. Evaluation was based on the “two out of three” rule, wherein at least two of the three blinded board‐certified dermatologists independently rated the post‐treatment images as improved compared to baseline. The lower bound of the 95% confidence interval for both analysis populations exceeded the pre‐specified threshold of 70%, confirming that the treatment met the efficacy criteria (FAS: *p* = 0.0015; PPS: *p* = 0.00045). This result indicates that the observed efficacy exceeds the pre‐specified performance threshold of 70%. Individual reviewer improvement rates were 85.37% (Reviewer 1), 87.80% (Reviewer 2), and 85.37% (Reviewer 3).

**TABLE 2:** Reviewer‐specific efficacy results in the per‐protocol set (PPS).

PPS 41 subjects	Effective counts	Efficacy	Standard error	95% Confidence lower limit	95% Confidence upper limit
Reviewer 1	35	85.37%	5.52%	70.83%	94.43%
Reviewer 2	36	87.80%	5.11%	73.80%	95.92%
Reviewer 3	35	85.37%	5.52%	70.83%	94.43%
Majority (≥ 2/3)	38	92.68%	4.07%	80.08%	98.46%

To characterize the magnitude of FWSS change beyond the binary responder endpoint, the underlying overall FWSS scores were summarized descriptively (Table 3). Median [Q1–Q3] baseline scores were 4 [4–4], 5 [4–6], and 5 [5–6] for Reviewers 1–3, respectively, and decreased at follow‐up to 3 [3–3], 3 [2–3], and 3 [3–4]. Mean score improvements were 1.12 ± 0.95, 2.15 ± 1.33, and 2.05 ± 1.95 points, respectively. Based on the median change across the three blinded evaluators for each participant, 10 subjects (24.4%) showed a 1‐point improvement, 21 (51.2%) a 2‐point improvement, and 7 (17.1%) an improvement of ≥ 3 points; 2 (4.9%) were unchanged and 1 (2.4%) worsened. These descriptive analyses supplement the prospectively specified primary responder endpoint and do not alter the two‐of‐three majority rule. Because the protocol specified a single overall FWSS assessment for each participant, separate region‐specific FWSS scores were not prospectively collected.

**TABLE 3:** Distribution and magnitude of changes in Fitzpatrick Wrinkle Severity Scale (FWSS).

Measure	Reviewer 1	Reviewer 2	Reviewer 3
Baseline FWSS, median [Q1–Q3]	4 [4–4]	5 [4–6]	5 [5–6]
Follow‐up FWSS, median [Q1–Q3]	3 [3–3]	3 [2–3]	3 [3–4]
Mean score improvement ± SD	1.12 ± 0.95	2.15 ± 1.33	2.05 ± 1.95
**Majority‐supported FWSS change**	***n* (%)**		
1‐point improvement	10 (24.4%)		
2‐point improvement	21 (51.2%)		
≥ 3‐point improvement	7 (17.1%)		
No change	2 (4.9%)		
Worsening	1 (2.4%)		
≥ 1‐point responder	38 (92.7%)		

*Note:* Data are based on the 41 participants in the PPS. FWSS improvement = baseline score − follow‐up score; positive values indicate improvement. Majority‐supported change is the median of the three evaluator‐specific FWSS changes for each participant and therefore reflects the magnitude of change supported by at least two evaluators. Q1, first quartile; Q3, third quartile; SD, standard deviation.

Inter‐rater agreement was additionally assessed for the binary responder classification. Across the three blinded evaluators, 106 of 123 individual classifications (86.2%) indicated a ≥ 1‐point FWSS improvement. Mean pairwise observed agreement for binary responder status was 77.2% (Reviewer 1 vs. Reviewer 2, 82.9%; Reviewer 1 vs. Reviewer 3, 75.6%; Reviewer 2 vs. Reviewer 3, 73.2%). Gwet's AC1 was 0.70, providing a chance‐corrected measure of agreement that is less affected by the high prevalence of positive responder classifications.

Representative before‐and‐after photographs were compared in Figure 3, demonstrating visible improvements following treatment with the micro‐radiofrequency device (nebulyft). For the first subject (top row), notable reductions in skin dullness and fine lines are observed, particularly along the nasolabial folds and periorbital areas. Skin texture appears smoother, with improved luminosity. For the second subject (bottom row), visible improvements are evident, including softening of wrinkles around the nasolabial crease and overall improvement in skin tone uniformity. These photographic outcomes align with the objective evaluator assessments and subject‐reported satisfaction, supporting the treatment's efficacy in enhancing skin appearance with minimal intervention.

**FIGURE 3:**
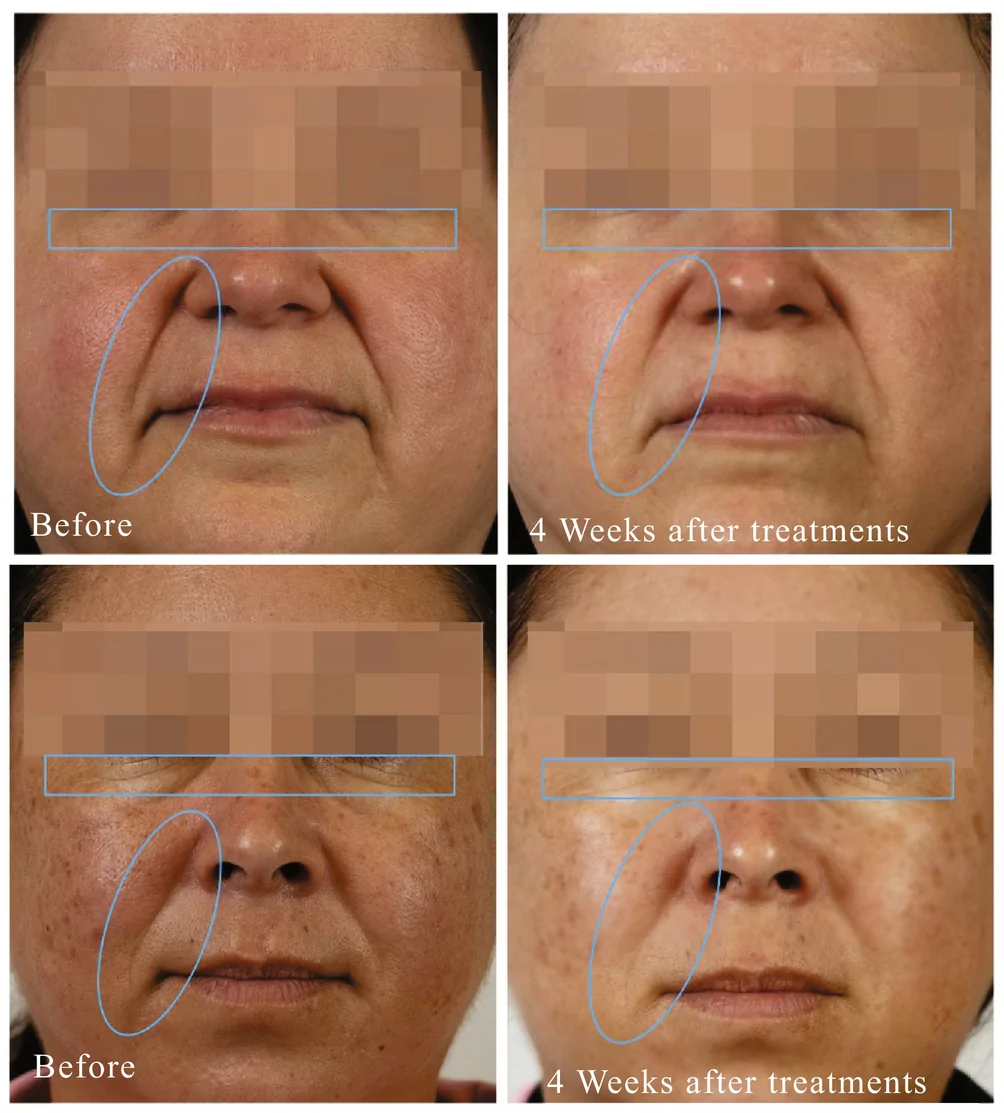
Before and after photographs of two representative cases, showing the improvement in skin's texture, wrinkles, and skin tone following treatment with the nebulyft microRF technology.

Subject‐reported outcomes were assessed using a standardized satisfaction questionnaire administered at the final follow‐up visit. Among the 41 participants who completed the study, the mean overall satisfaction score was 15.76 ± 0.54 out of a maximum score of 16. The median score was 16, with a range of 14–16, indicating high levels of satisfaction across all four assessed domains: treatment results, safety, ease of use, and ease of learning. These findings support strong subjective acceptance and perceived efficacy of the device among users.

All 42 enrolled subjects were included in the safety analysis set. No device‐related adverse events were reported during the study. Subjects did not report pain, skin irritation, or other treatment‐related complications during or after treatment sessions. Pain was assessed using a 0–10 Visual Analog Scale (VAS), and no subject rated the pain above zero during any session. No abnormal changes in vital signs were observed at follow‐up visits. The treatment was well tolerated, and all participants completed the treatment protocol without discontinuation due to safety concerns. No device malfunctions or labeling comprehension issues were reported.

## 4 Discussion

Previous RF literature has proposed collagen‐related tissue‐remodeling mechanisms and has reported that clinical improvement may continue after completion of treatment [24, 25]. The short‐term photographic improvements observed at 1 month in the present study may provide a preliminary clinical signal consistent with RF‐associated dermal remodeling described in prior literature. However, collagen fibril contraction, dermal perfusion, extracellular‐matrix changes, fibroblast activation, and neocollagenesis were not directly assessed in this study. Accordingly, these findings should be considered suggestive rather than confirmatory evidence of the underlying biological mechanisms.

Despite the positive outcomes, we acknowledge the inherent limitations of our study's single‐arm, open‐label design. Without a sham‐control group, it is difficult to entirely distinguish the physiological effects of the micro‐RF energy from potential placebo responses or response biases, particularly regarding subjective satisfaction scores. Additionally, the “Hawthorne effect”—whereby participants may adhere more strictly to general skincare and lifestyle routines during a clinical trial—cannot be entirely ruled out as a contributing factor to the observed improvements. Treatment adherence was prospectively documented using participant‐maintained daily diaries rather than device‐memory logs. Although this structured diary approach provided contemporaneous records of home use, some reporting bias cannot be completely excluded.

While this study provides prospective evidence supporting the safety and preliminary efficacy of 2‐W low‐power MEMS‐RF technology, these limitations highlight the need for further validation. Future research should prioritize randomized, sham‐controlled, double‐blinded trials with extended follow‐up periods (3–6 months). Such longitudinal studies will be important to isolate the specific therapeutic contribution of the device, assess the durability of the observed clinical improvements, and investigate the underlying biological tissue responses using objective structural or biological measurements.

As summarized in Table 4, most home RF devices intended for daily or near‐daily use operate at low‐to‐moderate output levels (~5.22–12 W) [24, 26]. A systematic review recommended every‐other‐day treatments at 10–12 W for 1–2 months [27]. By contrast, other devices report substantially higher power settings with lower treatment frequency—for example, a TriPollar protocol used weekly treatments at 37.5 W [28].

**TABLE 4:** Comparison of the RF skin care device.

Device and reference	Type and mode	Power level
nebulyft (this study)	Home‐use micro‐RF	2 W
NEWA 3DEEP [24]	Home‐use multi‐source phase RF	12 W
AMIRO ABF202 [26]	Home‐use bipolar RF (daily/deep)	5.22–6.25 W
Tripolar device [28]	Weekly used device	1 MHz, 37.5 W

Higher RF power generally entails greater risk of thermal injury and discomfort; importantly, clinical efficacy is not contingent upon using high power. The present study demonstrated that the micro‐radiofrequency device nebulyft (RT‐BA‐I) produced significant wrinkle reduction outcomes with a primary FAS responder rate of 90.48% (supportive PPS, 92.68%) evaluated by blinded dermatologists and an exceptionally high user satisfaction score (mean 15.76 out of 16). Notably, no adverse events or treatment‐related discomfort were reported, even with a high‐frequency usage of 28 sessions over 4 weeks. These results highlight that clinically meaningful aesthetic improvements can be achieved using high efficiency, low heat generating, coherent RF energy, suggesting that higher power may not be necessary for clinical efficacy.

This finding is consistent with the findings of Gold et al. [25], who reported that subsequent studies showed even modest increases in energy and treatment overlaps could heighten patient discomfort without necessarily improving clinical results. Electrode size and geometry may also influence RF energy distribution and penetration [29]. Tissue impedance should likewise be considered during device optimization. These considerations support further investigation of whether energy precision and targeted delivery, rather than absolute output power alone, contribute to clinical performance. The observed clinical improvement is compatible with biological responses previously proposed for RF treatment, including collagen‐related remodeling; however, fibroblast activation and collagen synthesis were not directly measured in the present study.

In conclusion, this prospective study demonstrated short‐term photographic improvement in facial wrinkles following treatment with the 2‐W MEMS‐based RF device, together with favorable tolerability and no device‐related adverse events observed during the study period. These findings provide preliminary clinical evidence supporting the feasibility of low‐power, phase‐controlled RF treatment. The observed improvement may be consistent with RF‐associated dermal remodeling described in previous studies; however, the present study did not directly assess biological tissue changes, and mechanistic conclusions cannot be drawn from these clinical findings alone. Longer‐term controlled studies incorporating objective structural or biological assessments are warranted.

## Author Contributions

Tony Shum performed the design of the protocol, supervised the clinical trial and the subjective evaluation. Aditi A. Sharma conducted the blinded assessment and discussion of the study. Shengshuo Yan, Rongwei Mao and Yang Zhang drafted the manuscript.

## Funding

The authors have nothing to report.

## Ethics Statement

This study was approved by the Advarra Internal Review Board in May 2024. All subjects in the study had signed the informed consent form. The authors confirm that the ethical policies of the journal, as noted on the journal's author guidelines page, have been adhered to and the appropriate ethical review committee approval has been received.

## Consent

All subjects had approved the use of their photos for the study.

## Conflicts of Interest

The authors declare no conflicts of interest.

## Data Availability

The data that support the findings of this study are available from the corresponding author upon reasonable request.
